# Parental income gradients in child and adolescent mortality: Norwegian trends over half a century

**DOI:** 10.1177/14034948231151990

**Published:** 2023-02-13

**Authors:** Miriam Evensen, Søren Toksvig Klitkou, Mette Christophersen Tollånes, Pétur Benedikt Júlíusson, Øystein Kravdal

**Affiliations:** 1 Institute for Social Research, Norway; 2 Clinical Trials Unit, Oslo University Hospital, Norway; 3 Department for Disease Burden, Norwegian Institute of Public Health, Norway; 4 Norwegian Organization for Quality Improvement of Laboratory Examinations (Noklus), Haraldsplass Deaconess Hospital, Norway; 5 Department for Global Public Health and Primary Care, University of Bergen, Norway; 6 Department of Health Registry Research and Development, Norwegian Institute of Public Health, Norway; 7 Department of Clinical Science, University of Bergen, Norway; 8 Children and Youth Clinic, Haukeland University Hospital, Norway; 9 Centre for Fertility and Health, Norwegian Institute of Public Health, Norway; 10 Department of Economics, University of Oslo, Norway

**Keywords:** Cause-specific mortality, children, adolescents, parental income, welfare state

## Abstract

**Background:**

Child mortality has declined rapidly over the last century in many high-income countries. However, little is known about the socio-economic differences in this decline and whether these vary across causes of death.

**Methods:**

We used register data that included all Norwegian births between 1968 and 2010 (2.1 million), and we analysed how all-cause and cause-specific child (0–4 years) and adolescent (5–20 years) mortality rates vary with relative parental income the year before the birth.

**Results:**

Child and adolescent all-cause mortality decreased with increasing parental relative income within all birth cohorts. Among children aged 0–4 years, the socio-economic gradient in all-cause mortality and in mortality due to external causes, sudden infant deaths and perinatal factors declined over the period, while there was no systematic decline in mortality from congenital malformations. Among children aged 5–20 years, the gradient did not weaken similarly, although there were indications of declines in the socio-economic gradient related to all-cause deaths and deaths because of suicides and other external causes. While the absolute differences in mortality declined over time, the relative differences remained stable.

**Conclusions:**

**Although children of low-income parents still have elevated mortality, there has been a large reduction in child mortality in all socio-economic groups across 50 years for all causes combined and most of the groups of specific causes of death.**

## Introduction

Social differences in children’s health have been reported in a large number of studies from both rich and poor countries. Most aspects of children’s health are positively linked to the parents’ socio-economic resources, and children born into socially advantaged families have the lowest mortality [1–5]. However, little is known about how the associations between childhood mortality and socio-economic status have changed over time. The few investigations of infant mortality that have addressed this have pointed in different directions, and there is hardly any statistical evidence regarding older children [6–10]. Also, it is not theoretically obvious what one should expect. On the one hand, the rising economic inequality documented in several countries, including Norway [11,12], may strengthen the social gradient in mortality, depending on how it is measured. On the other hand, introduction of medical technologies that can be widely applied without regard to individual income or knowledge, such as advances in neonatology in settings with public health care, may reduce mortality and leave the social gradient unchanged or even diminished.

One problem in such research is that it is challenging to measure parental socio-economic status consistently over time because of the secular changes in the distributions of educational and occupational attainments. Furthermore, vital statistics from many countries are not linkable to measures of individual socio-economic status. So, much current research on the topic has relied on area-level socio-economic indicators [10,13,14].

In Norway, registers that cover the entire population include individual-level data on births, deaths and socio-economic resources such as earnings. The current study examines how child mortality varies with socio-economic resources, as indicated by the parents’ ranking in the income distribution and the changes in this income gradient over 50 years. A relative income measure is used because of the great increase in earnings over the decades considered.

In addition to all-cause mortality, three or four causes of death are considered. We analyse children aged 0–4 years and those aged 5–20 years separately, as the causes of death and underlying behavioural and other pathways are different. Infant mortality in Norway is lower than in most other OECD countries, while the mortality of older children is close to the average [15].

## Methods

### Study population

We used data from administrative registers covering the entire Norwegian population: the Population Register (PR), the National Register for Personal Taxpayers, the Medical Birth Register (MFR) and the Cause of Death Register (CDR). The registers include personal identifiers that allow linkage between them and between children and parents. Because annual income data are available from 1967 and we considered the parents’ income the year before the child was born, we included children born in Norway in 1968 or later. As the data cover the years up to 2015, the last cohort included in the analysis of 0–4 mortality is 2010, and the last cohort included in the analysis of 5–20 mortality (conditioned on survival <5) is 1994. We excluded children whose parents’ income was zero (0.22%). In addition, a few children emigrated (1.85%), but supplementary analysis showed that excluding them had no impact on our estimates. Supplemental Table AI shows descriptive statistics for the study population.

### Statistical analysis and measures

We calculated the probability (per 1000 or 10,000) of dying within the fifth birthday and, for those still alive at that time, the probability of dying within the 21st birthday by using information on birth month and year taken from the PR. The causes of death are coded in accordance with the International Classification of Diseases, using the 8th, 9th and 10th revisions. We considered four groups of causes among 0- to 4-year-olds: perinatal factors, congenital malformations, sudden infant death syndrome and external causes. For children aged 5–20, we examined cancer mortality and mortality from external causes, with a distinction between suicides (intentional) and other external causes (see Supplemental Table AII).

Income was measured as the sum of the mother’s and father’s reported pensionable labour incomes, including earnings from self-employment, in the calendar year before the child’s birth. This income was transformed into an income rank by comparing with the corresponding incomes of the parents of other children born the same year. Parental income was constructed without adjusting for the number of children and adults in the child’s household, since there was inadequate information about household composition in the PR. Death probabilities for income rank vigintiles (rank 0–5%, 5–10%, etc.) were calculated for different cohort groups: 1968–1979, 1980–1989, 1990–1999 and 2000–2010 when considering children aged 0–4, and 1968–1975, 1976–1983 and 1984–1994 for those aged 5–20. For each of these cohort groups, we also estimated the linear relationship between parental income rank and the child death probability in a regression model, and we did an analysis for all cohorts, with an income–cohort interaction term included in order to make inferences about the changes in the linear relationship over the cohorts.

In addition to considering this linear relationship, which tells us how much mortality changes on an absolute scale as the parental income increases from the lowest to the highest vigintile, it is reasonable to pay some attention to the relative size of this mortality change, that is, how large it is compared to the mortality in the lowest quintile. Patterns in absolute and relative mortality may differ markedly. For example, the mortality of the poor divided by that of the rich may be larger in one cohort group than another, even though the absolute difference between them is the same. We define a ‘relative linear relationship (or trend)’ as the linear relationship (the slope in the regression) divided by the corresponding regression constant term (corresponding to mortality at the lowest income rank). All analyses were performed with STATA v16 (StataCorp, College Station, TX; see Appendix for methodological details).

## Results

### All-cause mortality among children aged 0–4 years

**Figure 1.**
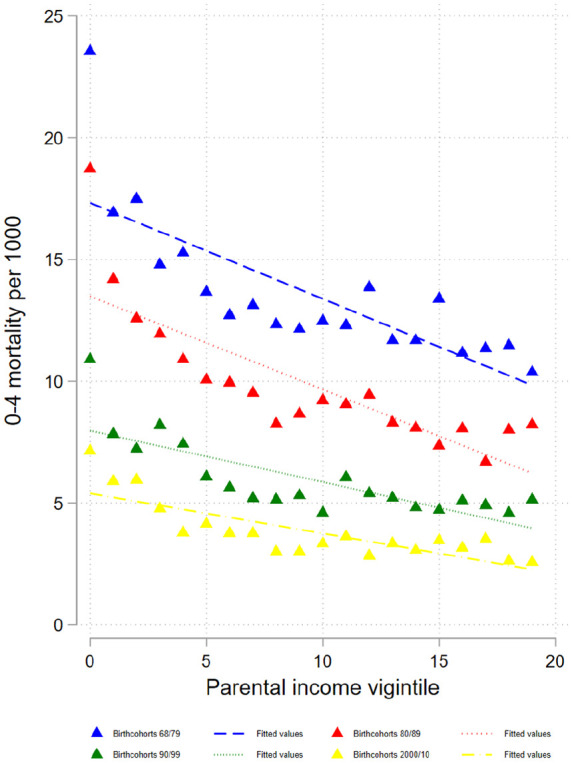
Probabilities of dying at age 0–4 years by parental income vigintiles for different birth cohorts. Note: Probabilities of dying are plotted across lowest to highest parental income vigintile (5% groups). Straight lines refer to linear fit.

Our sample includes 2,478,772 children who were observed up to five years of age, 21,094 of whom died within those five years. Moreover, 1,463, 869 children were observed up to 21 years of age, and 7125 of these died before their 21st birthday (see Supplemental Table AI for details). All-cause mortality among 0- to 4-year-olds declines with increasing parental relative income, as measured by vigintiles, in each of the four cohort groups (Figure 1). Supplemental Table AIII shows the linear trends as well as the corresponding constant terms in the regression and the relative linear trends. There is a negative linear trend for all four cohorts, but these trends are significantly weaker for the two last cohorts (see cohort interactions in Supplemental Table AV). In contrast, there is a less clear pattern in the development of the relative linear trend across cohorts.

### Cause-specific mortality among children aged 0–4 years

**Figure 2.**
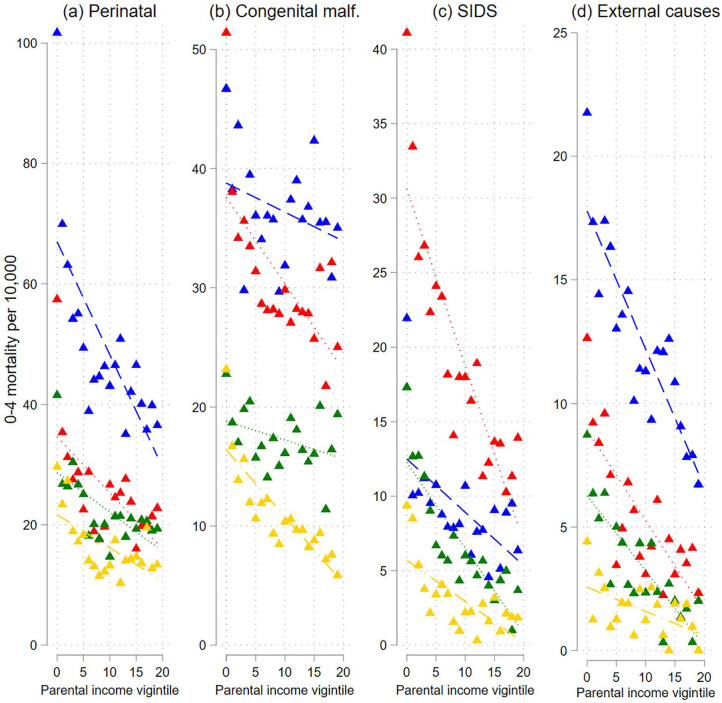
Probabilities of dying of selected causes (of death) at age 0–4 years by parental income vigintiles for different birth cohorts Note: Probabilities of dying are plotted across lowest to highest parental income vigintile (5% groups). Blue lines: 1968–1979; red lines: 1980–1989; green lines: 1990–1999; yellow lines: 2000–2010. Perinatal: certain conditions originating in the perinatal period; congenital malf.: congenital malformations and chromosomal abnormalities; SIDS: sudden infant death syndrome; external causes: external causes of injury and poisoning.

As with overall mortality, the risk of dying from perinatal causes decreases with increasing parental income in all cohorts (Figure 2(a), and Supplemental Table AIII(b)). The linear trend is weaker for each cohort, although the weakening is by far greatest from the oldest cohorts and much smaller across cohorts 1980–1989, 1990–1999 and 2000–2010. The relative trend is stable across the cohort groups. The risks of dying from congenital malformations (panel (b)) and SIDS (panel(c)) also decrease with higher income, but for the former, the linear trend does not change in a systematic way over the cohorts. The linear trend becomes gradually weaker over the three youngest cohort groups for SIDS (and the relative linear trend becomes stronger). Also, the risk of deaths due to external causes decreases with increasing parental income in all cohort groups (panel (d)). This relationship becomes weaker and is hardly visible in the latest cohort group, for whom the overall level of mortality from external causes is very low. The all-cause mortality level and the distribution of the causes of death differ between infants and children aged 0–4. We therefore also estimated the all-cause and cause-specific models specifically for infants (0–11 months). The patterns were very similar to those reported for children aged 0–4 years (see Supplemental Figures A1 and A2).

### Mortality among children aged 5–20 years

**Figure 3.**
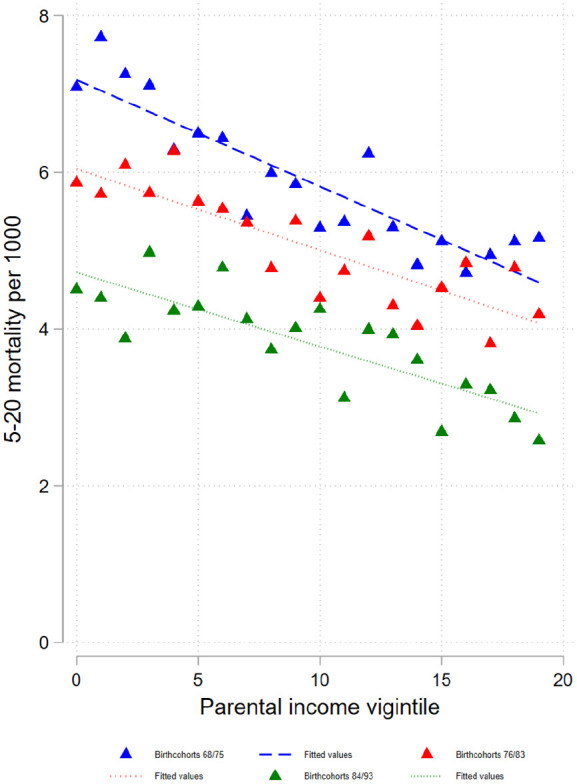
Probabilities of dying at age 5–20 years by parental income vigintiles for different birth cohorts. Note: Probabilities of dying are plotted across lowest to highest parental income vigintile (5% groups). Straight lines refer to linear fit.

Mortality among 5- to 20-year-olds is inversely related to parental income in all cohort groups: 1968–1975, 1976–1983 and 1984–1993 (Figure 3 and Supplemental Table AIV), and the point estimates of the interaction between cohort and parental income indicate that this relationship has become weaker over time (Supplemental Table AVI). The relative linear trend does not change systematically.

**Figure 4.**
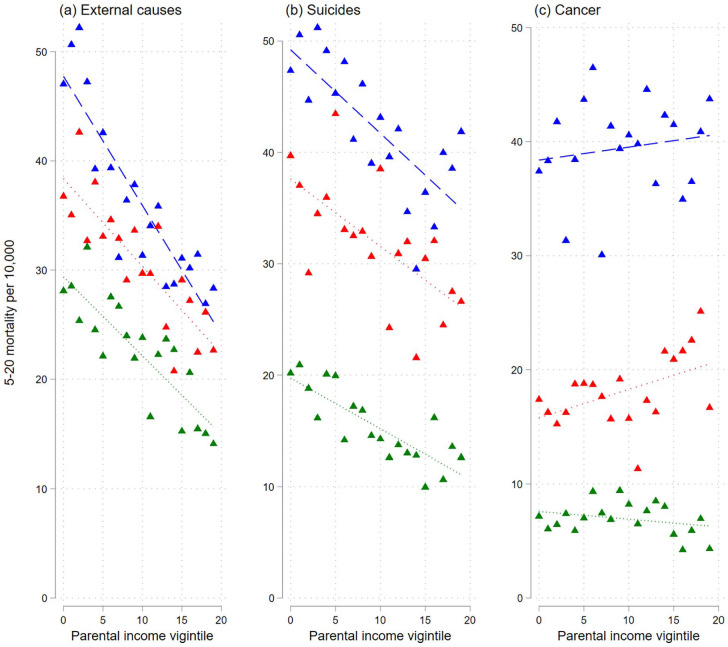
Probabilities of dying of selected causes (of death) at age 5–20 years by parental income vigintiles for different birth cohorts. Note: Probabilities of dying are plotted across lowest to highest parental income vigintile (5% groups). Straight lines refer to linear fit. Blue lines: 1968–1975; red lines: 1976–1983; green lines: 1984–1993. External causes: external causes of injury and poisoning (minus suicides).

The mortality from external causes of death or suicides also declines with increasing parental income. Again, the point estimates suggest a weakening relationship across the cohorts (Figure 4 and Supplemental Table AIV). There is a less clear development in the relative linear trends. The pattern is markedly different for cancer mortality, where an upward instead of downward gradient appears for the two oldest cohort groups.

## Discussion

Using population-wide data covering the last half century, this study shows that all cause-specific mortality among Norwegian children was inversely linked to the parents’ relative income rank. The negative association between all-cause mortality and parental income probably reflects that several factors underlying childhood mortality are affected by the parents’ purchasing power or other characteristics linked to income, such as maternal age and educational achievements. Examples of more proximate factors are mothers’ nutrition and smoking habits (with implications, for example, for the birth weight), whether the parents seek professional help when the child is sick and whether they otherwise care well for the child. In countries without a public health-care system, the association between income and use of high-quality health care may be stronger than in Norway. However, some differences in health-care utilisation among socio-economic groups are also apparent in Norway [16,17]. Additionally, an observed relationship between parents’ income and child mortality reflects that both these factors are influenced by the parents’ health in earlier years and several other individual and community characteristics.

For children younger than five years of age, the association between parents’ income and perinatal mortality, external causes of deaths or (except for the oldest cohort) SIDS has become weaker, while there is no such clear trend in the mortality from congenital malformations. The income gradient in all-cause mortality (which also includes deaths from other causes than the four groups that are considered) has also become weaker. It should be noted, however, that this holds for the absolute mortality differences. The relative linear trend, which is a measure of the absolute mortality difference (trend) compared to the mortality level among the poorest, has developed in a more irregular way, except that it has become more negative for SIDS.

The probability of dying from an external cause is influenced by the parents’ protective efforts as well as various preventive public measures, such as road safety, more use of seat belts in cars, helmets when cycling, stricter safety requirements regarding public playgrounds and dissemination of knowledge about home safety [18]. The general reduction of deaths due to external causes and the weakening of the income gradient has been consistent with a situation where (a) parents’ protection is positively linked to income or its correlates, such as education, (b) public preventive measures are strengthened and (c) preventive measures matter more for children with parents providing less protection (i.e. an interaction between these two factors). For example, children from low-income families may be more likely to live or attend schools in deprived areas with higher traffic volumes or substandard housing, which makes these children more exposed to hazards [19]. Public preventive measures such as regulations to separate car traffic from playground areas, reduce speed and develop safer pedestrian areas may then matter more for low-income children.

An argument similar to that for external causes of death may apply for SIDS when comparing among the three youngest cohort groups. Many countries witnessed relatively high SIDS rates in the 1980s and 1990s, which was related to advice about putting infants to sleep on their stomachs. During the early 1990s, official guidelines recommended parents to let their infants sleep on their back. This has been shown to be especially important for infants born preterm and with low birth weight, which is a more common situation among low-income than high-income families [20]. The information about sleep position provided to the public in the 1990s helped to almost eliminate this cause of death and made the earlier advantage that the well-resourced may have had (e.g. in terms of average better infant health and birth weight) gradually less relevant. However, social differences exist, even in the youngest cohorts. It is likely that SIDS mortality among recent cohorts is more linked to other factors than sleeping positions, such as maternal smoking and respiratory or gastrointestinal infections [21]. For this cause of death, the probability of dying was also higher among later-born cohorts than those born between 1967 and 1979. This likely reflects that SIDS did not have a unique ICD code until 1979, even though it was first defined in 1969 [22].

Advances in neonatal medical diagnostics and treatment, plus possibly an increasing tendency to terminate pregnancies because of information about serious defects, have contributed to a reduced mortality from congenital malformations. The reduction has been largely the same for all income groups (as judged by the lack of a systematic weakening of the income gradient over the half century that is considered). This is as one would expect if some sub-causes were eliminated by the introduction of new medical technology and everyone had similar access to this technology. Deaths from perinatal conditions – occurring usually within the first year of life – are largely related to low birth weight and low gestational age. The chance of premature birth is in turn influenced by, for example, maternal smoking and poor nutrition, which has typically been more common in families with a low income or low education [6,23]. Therefore, in a hypothetical situation where smoking is almost eliminated in all population groups, mortality from perinatal conditions would go down and the income gradient would become weaker. However, this is not what has happened: smoking rates have fallen more strongly among the socio-economically advantaged mothers [24]. Also, the intake of folic acid and other vitamins is still higher in the high-income groups [25]. In other words, the development in the intake of such supplements and smoking is not consistent with the change in the income gradient. Possibly, perinatal and obstetric care provided to everyone has generally increased the survival of relatively frail foetuses and children, thereby reducing the income gradient in mortality.

Among children aged 5–20, the relationship between income and all-cause mortality has not been so clearly weakened, although there are indications in that direction. The same can be said about mortality from external causes, while the income gradient in cancer mortality can be described as having become less positive and eventually negative (according to the point estimates).

As mentioned, personal risk behaviour – which tends to be connected with parental socio-economic resources [26] – is a key determinant of deaths due to external causes [27]. An example of special relevance for the relatively old children is that those whose parents have low education or income, and therefore perhaps also have a relatively high tendency to live apart, may be more likely than their peers from more resourceful families to develop a drug or alcohol problem, which may lead to fatal accidents [28]. The quite constant association between parental relative income and mortality from external causes may reflect that the links between income and the mentioned behavioural factors have been rather stable over time.

Children with low-earning parents may suffer more from mortality due to suicide because of their own or their parents’ mental-health problems, which in turn may be partly linked with unstable family situations [29]. The rather stable income gradient in suicide mortality indicates a persistent importance of parental income for such factors.

Socio-economic status may be associated with certain cancer types through social patterning of risk factors such as birth weight, parental age and environmental factors, although the direction of the association may vary [30]. For example, previous research has found that the incidence of brain tumours is highest in socio-economically advantaged residential areas, whereas some types of leukaemia tend to occur more often in low socio-economic settings [31]. One reason why we see indications of a gradually weaker positive income gradient, which eventually becomes negative, may be the shift in the relative frequency of various malignancies. While brain tumours were the most frequent diagnosis for the oldest cohort, leukaemia was more prevalent for the younger ones.

### Strengths and limitations

The major strengths of our study are that (a) the analysis is based on high-quality register data for an entire national population; (b) it covers several decades; (c) it involves a measure of socio-economic resources that is particularly suitable for a study of long-term trends; and (d) it includes (unlike most earlier studies) different causes of death in addition to all-cause mortality. Finally, information on the cause of death or income is missing for <1% and 0.2% of the sample, respectively.

Changes in coding practice constitute a limitation because they make it difficult to understand the development with respect to SIDS. Furthermore, children born abroad are not included, since we do not know the parents’ income the year before birth. Importantly, the chance of losing a child in a certain year is influenced by the parents’ resources at that time and some years back, which reflects incomes over several previous years. However, our measure of parental income refers to one particular year (the year before birth), when the income may have been markedly different from that in earlier years. That said, it would not have been a good idea to consider parental income at, say, age five instead, as this might have been affected by an earlier child death (i.e. reverse causality).

Parental education is also known to be an important correlate of children’s mortality [32,33], but the strong educational expansion over time calls for a relative measure, and this would be difficult to construct because education is not a continuous variable such as income; only a few educational categories are defined. Another potential problem with the present study is that the parents may not live together. In that case, the resources from which the child benefits may be smaller than suggested by the sum of the mother’s and father’s income. One possible argument is that the increasing prevalence of union dissolution would make our income measure an increasingly poor indicator of the income rank in later years. In other words, the importance of the current purchasing power for child mortality would – especially for the youngest cohorts – be larger than indicated by our results based on the pre-birth income.

Moreover, the relationship between income and child mortality is not necessarily linear, even though a linear trend was calculated for simplicity. On the contrary, the graphs suggest, as one would expect, that increasing income matters less at the higher levels.

Obviously, we cannot claim that we have estimated causal effects of parental income; there are several joint determinants of parental income and child mortality that we have not controlled for (and indeed would be almost impossible to control for). Also, a causal effect of parental income on child mortality would operate through a number of biosocial factors, and we make no attempt to identify any of these mediators.

Although children whose parents are in the lowest part of the income distribution still have elevated mortality compared to children from higher income families, there has been a considerable reduction in child mortality and a weakening of a social gradient over the 50 years of study.

## Supplemental Material

sj-docx-1-sjp-10.1177_14034948231151990 – Supplemental material for Parental income gradients in child and adolescent mortality: Norwegian trends over half a century Supplemental material, sj-docx-1-sjp-10.1177_14034948231151990 for Parental income gradients in child and adolescent mortality: Norwegian trends over half a century by Miriam Evensen, Søren Toksvig Klitkou, Mette Christophersen Tollånes, Pétur Benedikt Júlíusson and Øystein Kravdal in Scandinavian Journal of Public Health
