# Supplementary material for: Parental income gradients in child and adolescent mortality: Norwegian trends over half a century
Source: Scand J Public Health. 2023 Feb 13;52(4):402–9. doi: 10.1177/14034948231151990 (PMC11179307; doi:10.1177/14034948231151990)
Supplement: sj-docx-1-sjp-10.1177_14034948231151990 – Supplemental material for Parental income gradients in child and adolescent mortality: Norwegian trends over half a century [file sj-docx-1-sjp-10.1177_14034948231151990.docx]

**SUPPLEMENTS:**

Supplementary materials for “Parental income gradients in child and adolescent mortality: Norwegian trends over half a century “.

This file includes details on coding of causes of death, estimates for all-cause and cause-specific mortality for infants, estimates for cohort changes and a specification of the estimated linear probability models.

**Supplementary tables and figures:**

Table A1: Descriptive statistics for children born in Norway 1968-2010.

Table A2: Selected causes of deaths and codes.

Table A3: All cause and cause-specific estimates for mortality among 0-4 yrs old.

Table A4: All cause and cause-specific estimates for mortality among 5-20 yrs old.

Table A5: Estimated differences in the linear association between all-cause and cause-specific child mortality, ages 0-4, and parental income vigintile by birth cohort.

Table A6: Estimated differences in the linear association between all-cause and cause-specific child mortality, ages 5-20, and parental income vigintile by birth cohort.

Figure A1: Probabilities of infant death (0-11 months) by parental income vigintiles for different birth cohorts.

Figure A2: Probabilities of dying of selected causes (of death) of infant death (0-11 months) by parental income vigintiles for different birth cohorts.

| Table A1: Descriptive statistics for children born in Norway 1968-2010 | | | | | | | | | | | | | |
| --- | --- | --- | --- | --- | --- | --- | --- | --- | --- | --- | --- | --- | --- |
| Panel A. Deaths 0-4 years | |  |  |  |  |  |  |  |  |  |  |  |  |
|  |  | Birth cohort 1968-79 | |  | Birth cohort 1980-1989 | |  | Birth cohort 1990-1999 | |  | Birth cohort 2000-2010 | |  |
| Total number of deaths | | 9,866 |  |  | 5,190 |  |  | 3,578 |  |  | 2,460 |  |  |
| Percent |  | 1.38 |  |  | 0.99 |  |  | 0.60 |  |  | 0.38 |  |  |
| Parental income (mean) | | 307,521 |  |  | 413,705 |  |  | 485,911 |  |  | 673,848 |  |  |
| St.dev |  | 156,426 |  |  | 204,624 |  |  | 302,092 |  |  | 410,707 |  |  |
| Individuals |  | 713,999 |  |  | 525,869 |  |  | 597,394 |  |  | 641,510 |  |  |
|  |  |  |  |  |  |  |  |  |  |  |  |  |  |
| Panel B. Deaths 6-21 years | |  |  |  |  |  |  |  |  |  |  |  |  |
|  |  | Birth cohort 1968-1975 | |  | Birth cohort 1976-1983 | |  | Birth cohort 1984-1993 | |  |  |  |  |
| Total number of deaths | | 2,953 |  |  | 2,050 |  |  | 2,122 |  |  |  |  |  |
| Percent |  | 0.59 |  |  | 0.51 |  |  | 0.38 |  |  |  |  |  |
| Parental income (mean) | | 283,426 |  |  | 379,746 |  |  | 438,861 |  |  |  |  |  |
| St.dev |  | 143,118 |  |  | 227,397 |  |  | 227,397 |  |  |  |  |  |
| Individuals |  | 498,991 |  |  | 405,327 |  |  | 559,551 |  |  |  |  |  |

| Table A2: Selected causes of deaths and codes |  |  |  |
| --- | --- | --- | --- |
| ICD-version: | ICD-10 (1996-) | ICD-9 (1986-95) | ICD-8 (1969-86) |
| Cancer | C00-C97 | 140-208 | 140-209 |
| Certain conditions originating in the perinatal period | P00-P96 | 760-779 | 760-779 |
| Congenital malformations and chromosomal abnormalities | Q00-Q99 | 740-759 | 740-759 |
| Sudden infant death syndrome | R95 | 798.0 | 795 and age <2 years |
| External causes of injury and poisoning | V01-Y89 | E800-E999 | E800-E999 |
| Suicide | X60-X84 | E950-E959 | E950-E959 |

|  | Table A3: All cause and cause-specific estimates for mortality among 0-4 yrs old | | | | |  |  |
| --- | --- | --- | --- | --- | --- | --- | --- |
|  |  | Constant | Slope | Lower 95% CI | Upper 95% CI |  | Relativ slope |
|  | (a) All-cause | (1) | (2) | (3) | (4) |  | (5) |
|  | 1968-1979 | 0.0176208 | **-0.0004003** | -0.0004472 | -0.0003533 |  | -0.022717470 |
|  | 1980-1989 | 0.0135607 | **-0.0003886** | -0.0004349 | -0.0003422 |  | -0.028656338 |
|  | 1990-1999 | 0.0080268 | **-0.0002145** | -0.0002484 | -0.0001805 |  | -0.026722978 |
|  | 2000-2010 | 0.0054198 | **-0.0001669** | -0.0001931 | -0.0001406 |  | -0.030794494 |
|  | (b) Certain conditions originating in the perinatal period | | | |  |  |  |
|  | 1968-1979 | 0.0068216 | **-0.0001912** | -0.0002195 | -0.0001628 |  | -0.028028615 |
|  | 1980-1989 | 0.0034734 | **-0.0000930** | -0.0001168 | -0.0000692 |  | -0.026774918 |
|  | 1990-1999 | 0.0028658 | **-0.0000652** | -0.0000860 | -0.0000444 |  | -0.022751064 |
|  | 2000-2010 | 0.0021637 | **-0.0000550** | -0.0000721 | -0.0000378 |  | -0.02541942 |
|  | (c) Congenital malformations and chromosomal abnormalities | | | |  |  |  |
|  | 1968-1979 | 0.0038560 | **-0.0000227** | -0.000047 | 0.0000015 |  | -0.005886929 |
|  | 1980-1989 | 0.0037283 | **-0.0000718** | -0.0000976 | -0.0000459 |  | -0.019258107 |
|  | 1990-1999 | 0.0018820 | **-0.0000159** | -0.0000342 | 0.0000024 |  | -0.008448459 |
|  | 2000-2010 | 0.0016328 | **-0.0000559** | -0.0000699 | -0.0000418 |  | -0.034235669 |
|  | (d) Sudden infant death syndrome | |  |  |  |  |  |
|  | 1968-1979 | 0.0012093 | **-0.0000349** | -0.0000468 | -0.0000229 |  | -0.028859671 |
|  | 1980-1989 | 0.0030866 | **-0.0001199** | -0.0001406 | -0.0000993 |  | -0.038845331 |
|  | 1990-1999 | 0.0012315 | **-0.0000577** | -0.0000692 | -0.0000463 |  | -0.046853431 |
|  | 2000-2010 | 0.0005674 | **-0.0000274** | -0.0000348 | -0.000020 |  | -0.048290448 |
|  | (e) External causes of injury and poisoning. | | |  |  |  |  |
|  | 1968-1979 | 0.0018235 | **-0.0000568** | -0.0000712 | -0.0000424 |  | -0.031148889 |
|  | 1980-1989 | 0.0008910 | **-0.0000365** | -0.0000475 | -0.0000256 |  | -0.040965208 |
|  | 1990-1999 | 0.0006270 | **-0.0000304** | -0.0000385 | -0.0000223 |  | -0.048484848 |
|  | 2000-2010 | 0.0002539 | **-0.0000097** | -0.0000151 | -0.0000043 |  | -0.038046475 |
|  | Note: columns 1 and 2 show the constant and slope (linear trend) when mortality is regressed on parental income rank with a linear probability model. Lower 95 per cent CI is given in column 3 and upper in column 4. Column 5 shows the relative slope defined as slope divided by constant. Coefficients in bold are significant at *P*<0.05 level. | | | | | | |
|  |  |  |  |  |  |  |  |
|  |  |  |  |  |  |  |  |


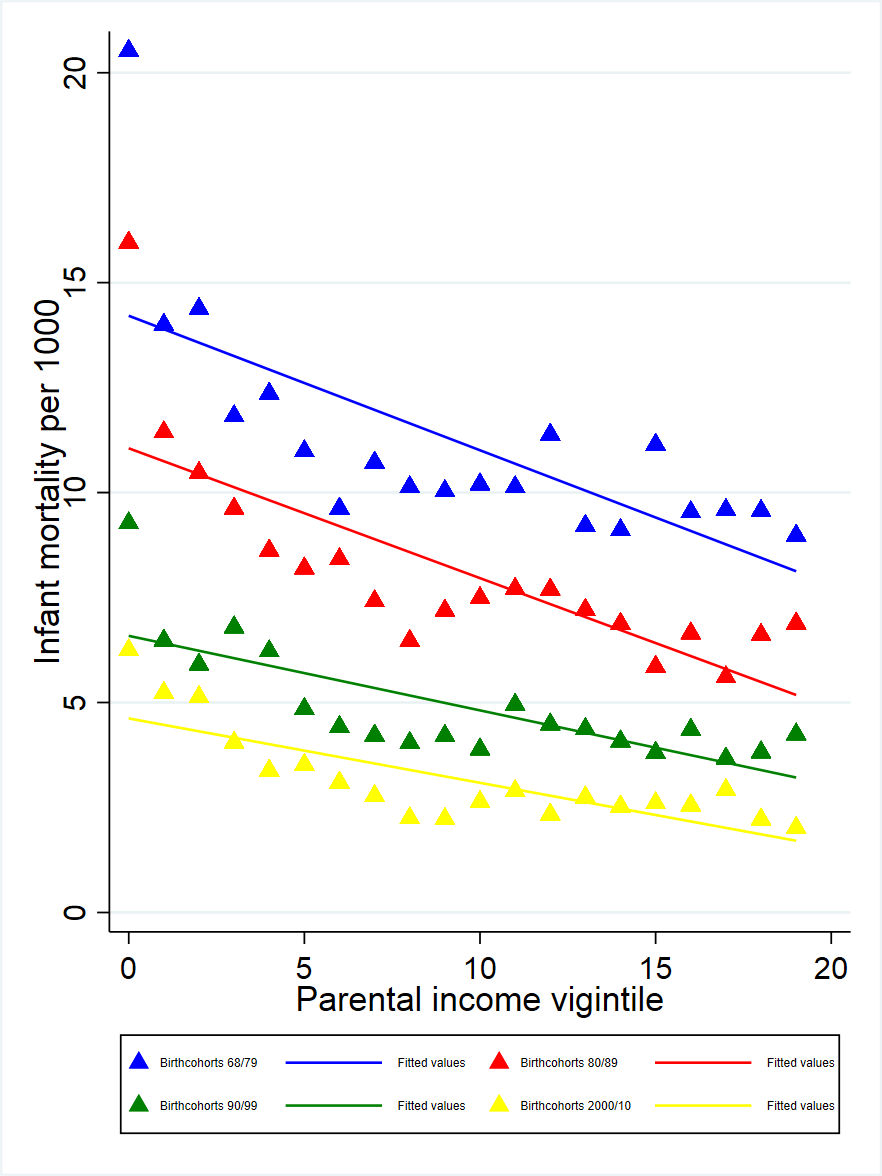


**Figure A1: Probabilities of infant death (0-11 months) by parental income vigintiles for different birth cohorts.**

Note: Blue lines:1968-1979, Red lines: 1980-1989, Green lines:1990-1999, Yellow lines: 2000-2010.


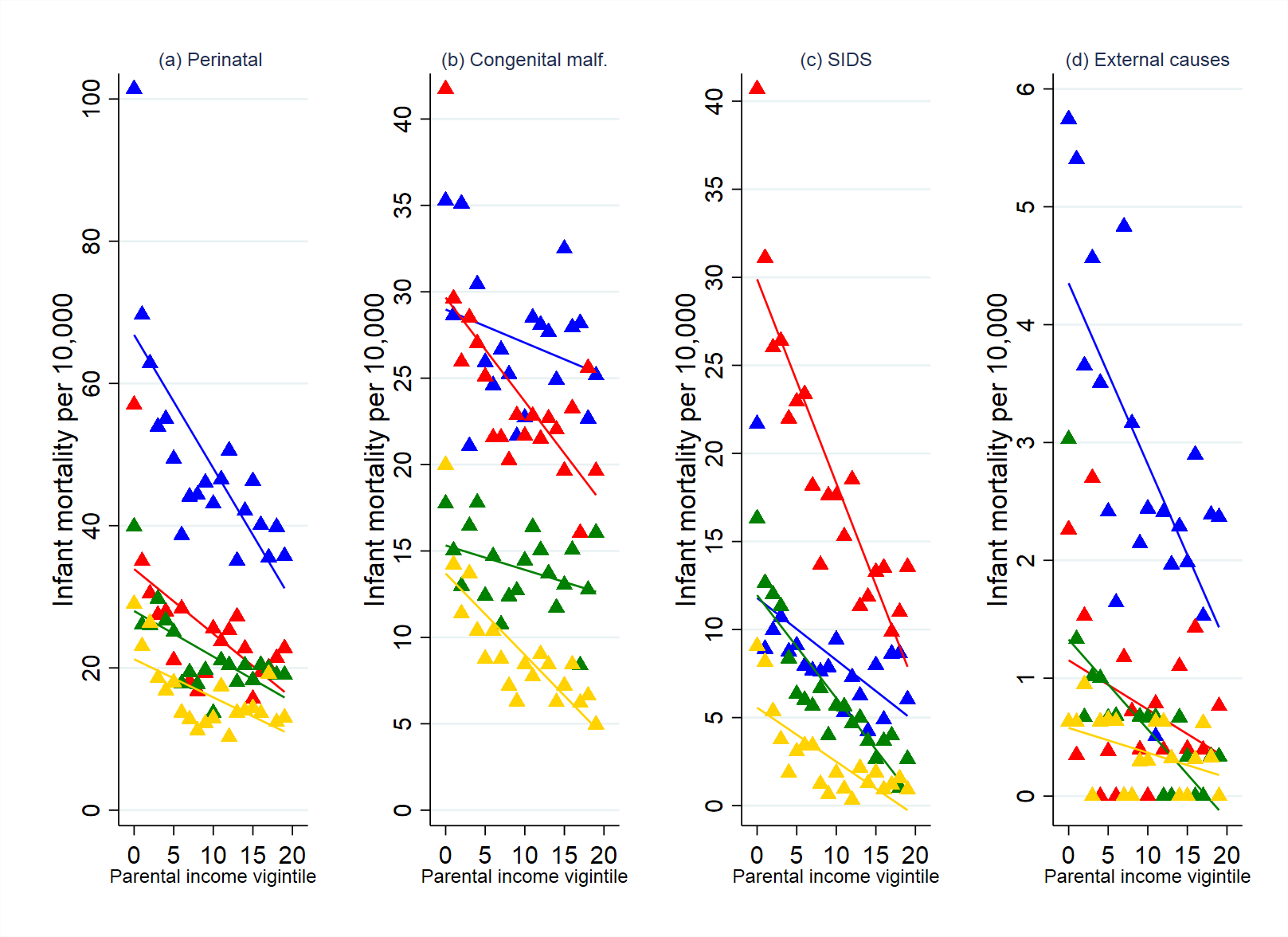


**Figure A2: Probabilities of dying of selected causes of infant death (0-11 months) by parental income vigintiles for different birth cohorts.**

Note: Blue lines:1968-1979, Red lines: 1980-1989, Green lines:1990-1999, Yellow lines: 2000-2010.

| Table A4:All-cause and cause-specific estimates for mortality among 5-20 year olds | | | | | | |
| --- | --- | --- | --- | --- | --- | --- |
|  | Constant | Slope | Lower 95% CI | Upper 95% CI |  | Relative slope |
| (a) All-cause | (1) | (2) | (3) | (4) |  | (5) |
| 1968-1975 | 0.0072115 | **-0.0001360** | -0.0001729 | -0.0000990 |  | -0.018858767 |
| 1976-1983 | 0.0060453 | **-0.0001038** | -0.0001417 | -0.0000660 |  | -0.017170364 |
| 1984-1993 | 0.0047099 | **-0.0000965** | -0.0001244 | -0.0000685 |  | -0.020488758 |
|  |  |  |  |  |  |  |
| (b) External causes (excluding suicides) | | | |  |  |  |
| 1968-1975 | 0.0036691 | **-0.0000815** | -0.0001073 | -0.0000556 |  | -0.022212532 |
| 1976-1983 | 0.0027284 | **-0.0000594** | -0.0000842 | -0.0000346 |  | -0.021771001 |
| 1984-1993 | 0.0020319 | **-0.0000509** | -0.0000688 | -0.0000330 |  | -0.025050445 |
|  |  |  |  |  |  |  |
| (c)Suicide |  |  |  |  |  |  |
| 1968-1975 | 0.0049323 | **-0.0000746** | -0.0001058 | -0.0000434 |  | -0.01512479 |
| 1976-1983 | 0.0037658 | **-0.0000605** | -0.0000907 | -0.0000304 |  | -0.016065643 |
| 1984-1993 | 0.0019310 | -0.0000446 | -0.0000623 | -0.0000270 |  | -0.023096841 |
|  |  |  |  |  |  |  |
| (d) Cancer |  |  |  |  |  |  |
| 1968-1975 | 0.0038820 | 0.0000116 | -0.0000188 | 0.0000419 |  | 0.00298815 |
| 1976-1983 | 0.0015828 | **0.0000250** | 0.0000022 | 0.0000478 |  | 0.015794794 |
| 1984-1993 | 0.0007525 | -0.0000073 | -0.0000192 | 0.0000045 |  | -0.009754153 |
| Note: columns 1 and 2 show the constant and slope (linear trend) when mortality is regressed on parental income rank with a linear probability model. Lower 95 per cent CI is given in column 3 and upper in column 4. Column 5 shows the relative slope defined as slope divided by constant. Coefficients in bold are significant at P<0.05 level. | | | | | | |
|  |  |  |  |  |  |  |
|  |  |  |  |  |  |  |

**Statistical models**

For all-cause and cause-specific measures of child mortality, we estimate the following linear regression model:

M *_i_*  =β_0_+β_1_Parental income *_i_*  +ε_i_  (1)

where *M_i_* is 1 if child i dies from the mortality outcome under study within the relevant observation period (age 0-4 or age 5-20) and otherwise is 0. *Parental income_i_* is the parental income rank, which is a number between 0 (the poorest parents) to 1 (the richest parents). The coefficient of interest is *β*_1_, which is referred to as the main text as the income gradient, or the linear relationship (trend) between relative income and mortality.

Estimates of  *β*_1_ and the corresponding 95% confidence intervals are shown in tables along with the relative linear relationships (relative slopes) defined as *β*_1_ divided by β_0_.

To find out whether the income gradient changes over cohorts, an interaction between Parental income and cohort group was added along with the cohort main effect. More specifically, the following model was estimated for all cohorts combined:

M *_i_*  =β_0_+β_1_ Parental income *_i_*  + β_2_ Parental income *_i_* * K_i_ + β_3_ K_i_ ε_i_  (2)

K_i_ is a categorical birth cohort variable with the oldest cohort group as the reference (i.e. when analysing mortality between ages 0 and 4, the birth cohort 1968-1979 serves as the reference group, and when the focus is on mortality between ages 5 and 20, 1968-1975 serves as the reference category).

The β_2_ coefficients are shown in tables A5 and A6.

| Table A5: Estimated differences in the linear association between all-cause and cause-specific child mortality, ages 0-4, and parental income vigintile by birth cohort (N=2,478,772). | | | | | | | | | | | | | | | | | | | | |
| --- | --- | --- | --- | --- | --- | --- | --- | --- | --- | --- | --- | --- | --- | --- | --- | --- | --- | --- | --- | --- |
|  |  | (a) All-cause mortality | |  |  | (b) Perinatal conditions | |  |  | (c) Congenital malformations | | |  | (d) Sudden infant death syndrome | | |  | (e) External causes | |  |
|  |  |  | Lower | Upper |  |  | Lower | Upper |  |  | Lower | Upper |  |  | Lower | Upper |  |  | Lower | Upper |
|  |  | Coef. | 95% CI | 95% CI |  | Coef. | 95% CI | 95% CI |  | Coef. | 95% CI | 95% CI |  | Coef. | 95% CI | 95% CI |  | Coef. | 95% CI | 95% CI |
| Vigintile (linear) | | -0.00040 | -0.00044 | -0.00036 |  | -0.00019 | -0.00021 | -0.00017 |  | -0.00002 | -0.00004 | 0.00000 |  | -0.00003 | -0.00005 | -0.00002 |  | -0.00006 | -0.00007 | -0.00005 |
| Cohorts (ref. = 1968-1979) | |  |  |  |  |  |  |  |  |  |  |  |  |  |  |  |  |  |  |  |
|  | Cohort 1980-1989 | -0.00406 | -0.00469 | -0.00343 |  | -0.00335 | -0.00372 | -0.00298 |  | -0.00013 | -0.00046 | 0.00021 |  | 0.00188 | 0.00167 | 0.00208 |  | -0.00093 | -0.00110 | -0.00076 |
|  | Cohort 1990-1999 | -0.00959 | -0.01020 | -0.00899 |  | -0.00396 | -0.00432 | -0.00360 |  | -0.00197 | -0.00230 | -0.00165 |  | 0.00002 | -0.00018 | 0.00022 |  | -0.00120 | -0.00136 | -0.00103 |
|  | Cohort 2000-2010 | -0.01220 | -0.01280 | -0.01160 |  | -0.00466 | -0.00501 | -0.00431 |  | -0.00222 | -0.00254 | -0.00191 |  | -0.00064 | -0.00084 | -0.00045 |  | -0.00157 | -0.00173 | -0.00141 |
| Interaction terms | |  |  |  |  |  |  |  |  |  |  |  |  |  |  |  |  |  |  |  |
|  | Vigintile x Cohort 1980-1989 | 0.00001 | -0.00004 | 0.00007 |  | **0.00010** | 0.00006 | 0.00013 |  | **-0.00005** | -0.00008 | -0.00002 |  | **-0.00009** | -0.00010 | -0.00007 |  | **0.00002** | 0.00000 | 0.00004 |
|  | Vigintile x Cohort 1990-1999 | **0.00019** | 0.00013 | 0.00024 |  | **0.00013** | 0.00009 | 0.00016 |  | 0.00001 | -0.00002 | 0.00004 |  | **-0.00002** | -0.00004 | 0.00000 |  | **0.00003** | 0.00001 | 0.00004 |
|  | Vigintile x Cohort 2000-2010 | **0.00023** | 0.00018 | 0.00029 |  | **0.00014** | 0.00010 | 0.00017 |  | **-0.00003** | -0.00006 | 0.00000 |  | 0.00001 | -0.00001 | 0.00003 |  | **0.00005** | 0.00003 | 0.00006 |
| Intercept | | 0.01762 | 0.01721 | 0.01803 |  | 0.00682 | 0.00658 | 0.00706 |  | 0.00386 | 0.00364 | 0.00407 |  | 0.00121 | 0.00107 | 0.00134 |  | 0.00182 | 0.00171 | 0.00193 |
| Note: Coefficients in bold are significant at he *P*<0.05-level. Abbreviations: Perinatal conditions, Certain conditions originating in the perinatal period; Congenital malformations, Congenital malformations and chromosomal abnormalities; External causes, External causes of injury and poisoning. | | | | | | | | | | | | | | | | | | | | |

| Table A6: Estimated differences in the linear association between all-cause and cause-specific child mortality, ages 5-20, and parental income vigintile by birth cohort (N=1,463,869). | | | | | | | | | | | | | | | | |
| --- | --- | --- | --- | --- | --- | --- | --- | --- | --- | --- | --- | --- | --- | --- | --- | --- |
|  |  | (a) All-cause mortality | | |  | (b) External causes | | |  | (c) Suicides | | |  | (d) Cancer | | |
|  |  |  | Lower | Upper |  |  | Lower | Upper |  |  | Lower | Upper |  |  | Lower | Upper |
|  |  | Coef. | 95% CI | 95% CI |  | Coef. | 95% CI | 95% CI |  | Coef. | 95% CI | 95% CI |  | Coef. | 95% CI | 95% CI |
| Vigintile (linear) | | -0.00014 | -0.00017 | -0.00010 |  | 0.00001 | -0.00008 | -0.00003 |  | -0.00006 | -0.00009 | -0.00003 |  | 0.00003 | 0.00000 | 0.00005 |
| Cohorts (ref. = 1968-1975) | |  |  |  |  |  |  |  |  |  |  |  |  |  |  |  |
|  | Cohort 1976-1983 | -0.00117 | -0.00172 | -0.00061 |  | 0.00094 | 0.00057 | 0.00131 |  | 0.00117 | 0.00074 | 0.00160 |  | 0.00230 | 0.00193 | 0.00267 |
|  | Cohort 1984-1993 | -0.00250 | -0.00301 | -0.00199 |  | -0.00070 | -0.00106 | -0.00033 |  | -0.00183 | -0.00225 | -0.00142 |  | -0.00083 | -0.00119 | -0.00047 |
| Interaction terms | |  |  |  |  |  |  |  |  |  |  |  |  |  |  |  |
|  | Vigintile x Cohort 1976-1983 | 0.00003 | -0.00002 | 0.00008 |  | -0.00002 | -0.00006 | 0.00001 |  | -0.00001 | -0.00005 | 0.00002 |  | -0.00001 | -0.00005 | 0.00002 |
|  | Vigintile x Cohort 1984-1993 | 0.00004 | -0.00001 | 0.00009 |  | 0.00001 | -0.00002 | 0.00004 |  | 0.00002 | -0.00002 | 0.00005 |  | **-0.00003** | -0.00006 | 0.00000 |
| Intercept | | 0.00721 | 0.00684 | 0.00758 |  | 0.00273 | 0.00245 | 0.00301 |  | 0.00377 | 0.00345 | 0.00409 |  | 0.00158 | 0.00131 | 0.00186 |
| Note: Coefficients in bold are significant at the *P*<0.05-level. Abbreviations: External causes, refers External causes of injury and poisoning minus suicides which is included as a separate cause of death. | | | | | | | | | | | | | | | | |
|  |  |  |  |  |  |  |  |  |  |  |  |  |  |  |  |  |
|  |  |  |  |  |  |  |  |  |  |  |  |  |  |  |  |  |
